# Beyond Ribosomal Binding: The Increased Polarity and Aberrant Molecular Interactions of 3-*epi-*deoxynivalenol

**DOI:** 10.3390/toxins8090261

**Published:** 2016-09-08

**Authors:** Yousef I. Hassan, Honghui Zhu, Yan Zhu, Ting Zhou

**Affiliations:** Guelph Research and Development Centre, Agriculture and Agri-Food Canada, Guelph, ON N1G5C9, Canada; yousef.hassan@agr.gc.ca (Y.I.H.); honghui.zhu@agr.gc.ca (H.Z.); yan.zhu@agr.gc.ca (Y.Z.)

**Keywords:** deoxynivalenol, epimer, polarity, Tri101, molecular, interactions

## Abstract

Deoxynivalenol (DON) is a secondary fungal metabolite and contaminant mycotoxin that is widely detected in wheat and corn products cultivated around the world. Bio-remediation methods have been extensively studied in the past two decades and promising ways to reduce DON-associated toxicities have been reported. Bacterial epimerization of DON at the C3 carbon was recently reported to induce a significant loss in the bio-toxicity of the resulting stereoisomer (3-*epi-*DON) in comparison to the parental compound, DON. In an earlier study, we confirmed the diminished bio-potency of 3-*epi-*DON using different mammalian cell lines and mouse models and mechanistically attributed it to the reduced binding of 3-*epi-*DON within the ribosomal peptidyl transferase center (PTC). In the current study and by inspecting the chromatographic behavior of 3-*epi-*DON and its molecular interactions with a well-characterized enzyme, *Fusarium graminearum* Tri101 acetyltransferase, we provide the evidence that the C3 carbon epimerization of DON influences its molecular interactions beyond the abrogated PTC binding.

## 1. Introduction

Deoxynivalenol (DON) is a secondary fungal metabolite and contaminant mycotoxin that is wildly detected in wheat and corn products cultivated around the world. It is estimated that more than four billion people are exposed to high levels of this metabolite especially in developing countries [1]. In mono-gastric animals, DON presence within feed is associated with adverse effects ranging from low animal-productivity, feed-refusal, to decreased weight-gain [2,3,4]. Similarly, DON causes both chronic and acute symptoms in humans with changes observed at the molecular and cellular levels [5,6]. Intestinal inflammations, increased susceptibility to infections, negative effects on the active transport of many nutrients [2,7], and brain homeostasis [8] are among the reported outcomes of DON exposure. Finally, DON is considered a major factor in the pathogenicity of Fusarium species against plants [9].

DON initial toxicity emerges from its ability to bind eukaryotic ribosomes hence inhibits protein biosynthesis [2,5,10]. DON binds to the 60S subunit and inside the A-site of the peptidyl transferase center (PTC) of the wildtype ribosome forming three hydrogen bonds: (a) The first is between the hydrogen of the uracil U2873 sugar and the oxygen of the epoxy group on C12 in DON; (b) The second is between the hydrogen of the guanine G2403 and the oxygen of the C15 group CH_2_OH in DON; (c) Finally, the oxygen of uracil U2869 interacts with hydrogen of the C3 group in DON [11,12]. The attenuation or abrogation of such interactions as in the case of ribosomal protein L3 (RPL3) mutants lead to a semi-dominant resistance toward this toxin in plants and yeast [13,14,15].

The chemical structure of DON influences both its potency and the associated sensitivity-levels endured in different animal species [2]. Changes to DON structure (such as de-epoxidation, acetylation, and hydroxylation) can substantially influence its toxicity [2,16]. For example, the C15 carbon acetylation is report to increase the toxicological potency of DON in Caco-2 cells while modifications that take place at the C3 carbon are reported to decrease its toxicity by a number of orders of magnitude [2].

Many microorganisms isolated in the past two decades including bacteria, filamentary fungi, and yeasts were shown to reduce DON toxicity [17,18,19] either by physically binding DON (hence reducing its absorption) or through introducing enzymatic/chemical modifications at specific side groups [20,21,22]. Recently, a soil bacterium identified as *Devosia mutans* Strain 17-2-E-8, was reported to epimerize the -OH group at the C3 carbon within DON leading to a significant abrogation of DON cellular toxicity [23,24]. The diminished toxicity of this epimer, 3-*epi-*DON, was confirmed in different cell lines including Caco-2 and 3T3 fibroblasts in addition to B6C3F1 mouse model [25]. Other teams have reported similar results for 3-*epi-*DON [26,27].

In spite of the confirmed reduction of toxicity for 3-*epi-*DON using different in vivo models, a clear mechanistic explanation for the drastic reduction in potency connected with just one (-OH) group rotation is lacking. A recent study compared the toxicological profiles of DON, 3-*epi-*DON, and deepoxy-deoxynivalenol (DOM-1) and confirmed the toxicity reduction for both 3-*epi-*DON and DOM-1 attributing the reduction of 3-*epi-*DON cellular toxicity to the reduced hydrogen bonding of 3-*epi-*DON within the ribosomal peptidyl transferase center [12].

In this study, we first compare the chromatographic behavior of DON and 3-*epi-*DON and later explore the ability of both compounds to interact with a recombinant *Fusarium graminearum* Tri101 acetyltransferase [28] by tracking acetyl-groups transfer as an indicative marker of such interactions. While the reduced toxicity of 3-*epi-*DON can be plausibly attributed to the attenuated binding of 3-*epi-*DON to ribosomal peptidyl transferase centers, the obtained data show that the increased polarity of 3-*epi-*DON in addition to its aberrant molecular interactions are indeed factors that can contribute to the overall toxicity reduction possibly in a synergistic fashion.

## 2. Results

### 2.1. Fusarium graminearum Tri101 Acetyltransferase Expression and Purification

**Figure 1:**
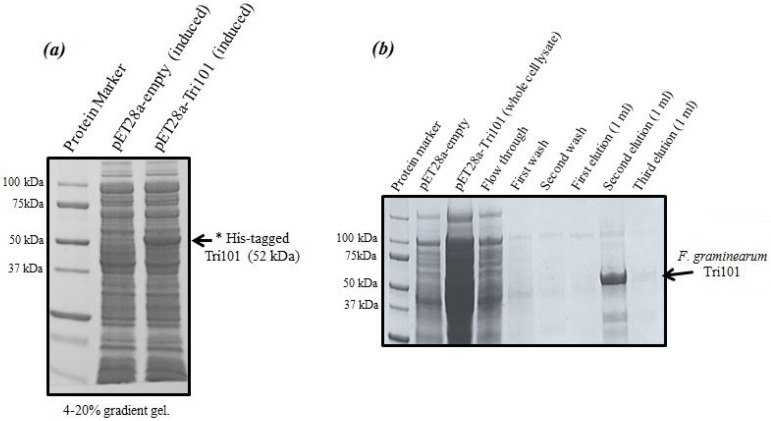
The induction and purification of His-FgTri101 expressed in *E. coli* BL21(DE3) cells. (**a**) His-FgTri101 from *Fusarium graminearum* was induced and overexpressed in BL21(DE3) cells. (**b**) The enzyme was purified with Clontech’s His-TALON gravity columns by overnight incubation at 4 °C. All washes and subsequent elution steps were conducted at room temperature.

Expression levels of His-tagged FgTri101 in *E. coli* whole cell lysates are shown in Figure 1a. A clear band of the induced enzyme with the expected molecular weight (52 kDa) was present in the third lane after 0.5 mM IPTG addition overnight.

It was critical to start the induction within the OD_600_ = 0.6–0.8 range as higher values led to protein degradation and much lower yields. The induction took place also at 16 °C overnight even though such low temperatures were not always necessary and the induction could possibly proceed at 37 °C. The outcome of His-FgTri101 purification is shown in Figure 1b. The second elution led to the least contaminated yet highest enzyme yield. Final protein preparations were subdivided in aliquots (10–20 µL) and stored at −20 °C until usage.

### 2.2. Chromatographic Elution Patterns Suggested the Increased Polarity of 3-epi-DON

**Figure 2:**
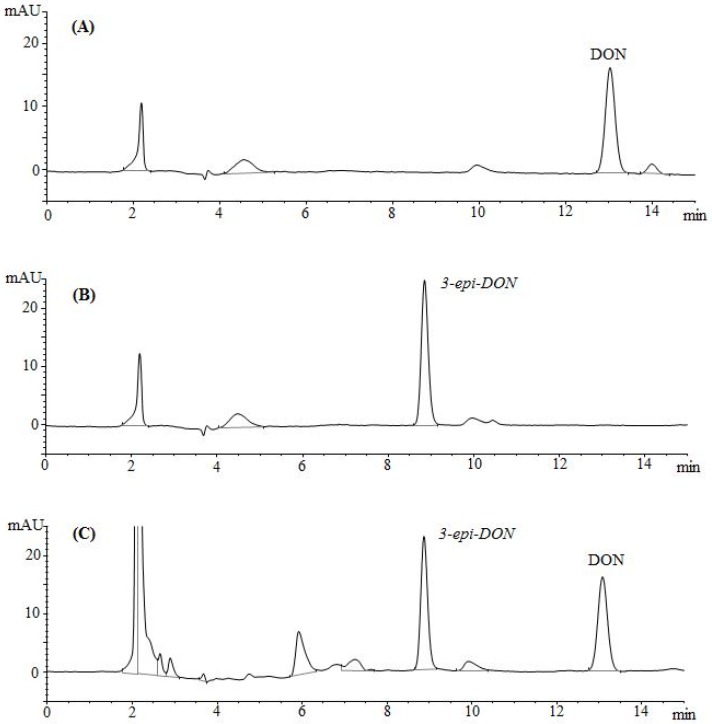
Chromatographic separation of DON and 3-*epi-*DON. Both DON and 3-*epi-*DON were injected on Jupiter 4µ Proteo 90A HPLC column. The results indicated that DON (less-polar) eluted in a longer time frame (**A**) in comparison to 3-*epi-*DON (**B**) under the same isocratic separation conditions. Section (**C**) represents the injection of both compounds at the same run.

The epimerization of DON at the C3 group rendered 3-*epi-*DON to a higher polarity spectrum compared to the parental compound, DON. Under the same isocratic separation conditions (buffer composition, temperature, reverse phase column), DON was originally eluting at 13.2 min. (Figure 2A) however after the bacterial epimerization, 3-*epi-*DON shifted to the 8.9 min. (Figure 2B). The noted change in elution time, despite the similarity in structures/molecular weights/ionization status, was an indicative of changes of the polarity index associated with electron rearrangements. Figure 2C evidently presents DON and 3-*epi-*DON separation patterns for *Devosia mutans* 17-2-E-8 LB broth sample under the same analytical conditions (isocratic) and using a reverse-phase C12 Jupiter 4µ Proteo 90A HPLC column (commonly used for the purification of peptide mixtures) which reflected an altered interactions of 3-*epi-*DON with the column matrix. While chromatographic separations are not conventionally used to draw conclusions about polarity changes of mycotoxins, the data presented here collectively supported the notions that the observed changes in elution times are due to the increased polarity of 3-*epi-*DON. Our experimental approach was similar to the one that was suggested earlier by Namjesnik-Dejanovic and Cabaniss [29].

### 2.3. Fusarium graminearum Tri101 Carried DON Acetylation Both in Vitro and in Vivo but Not 3-epi-DON

As mentioned below in the Materials and Methods section, FgTri101 was used to track the molecular interactions of DON and 3-*epi-*DON as it provides means of measuring such interactions indirectly and quantitatively. Both in vivo and in vitro models were used to minimize any aberrant interpretations resulting from DON and 3-*epi-*DON abilities to adsorb to bacterial cell walls/proteins hence reduce their availability within cellular matrixes/reaction mixes.

**Figure 3:**
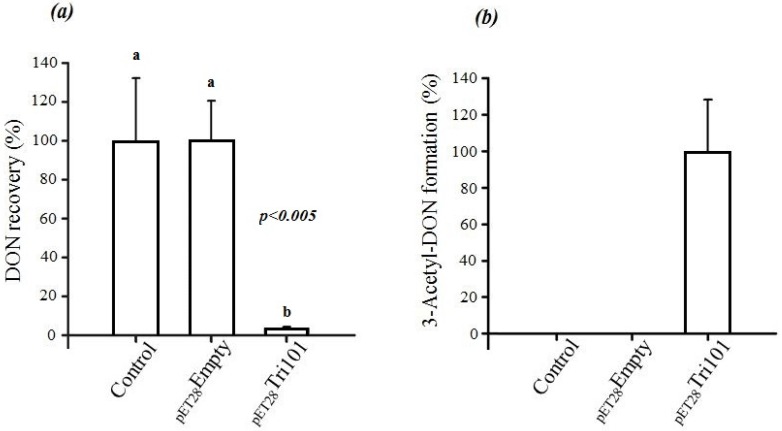
Changes in DON concentrations in *E. coli* BL21(DE3) cells overexpressing *Fusarium graminearum* Tri101. (**a**) A significant decrease in DON recovery was noted for the construct that encoded for the Tri101 enzyme in comparison to the control or the empty vector. The noted decrease in DON was confirmed to be due to the acetylation of DON by His-FgTri101 (**b**). The above tendency was observed in two separate experiments, each with three replications, and the different letters signify significant differences between the means according to Fisher’s Least Significant Difference (LSD) test (*p <* 0.005).

In short, FgTri101 was able to interact with DON both in vivo and in vitro as indicated by the C3 carbon acetylation. The in vivo interactions between DON and *E. coli* BL21(DE3) cells expressing His-FgTri101 led to a significant decrease of DON recovery (Figure 3a) in test tubes in comparison to the tubes that harbored only the LB broth supplemented with 50 µg/mL DON (control) or the empty pET-28a(+) vector (negative control) (Figure 3a).

Furthermore, FgTri101 presence with DON led to the accumulation of the 3ADON metabolite as shown in Figure 3b. No 3ADON was detected in tubes harboring LB broth (with 50 µg/mL DON) nor the empty pET-28a(+) vector (negative control) alone indicating that the accumulation was the result of specific DON/FgTri101 interactions (acetylation).

**Figure 4:**
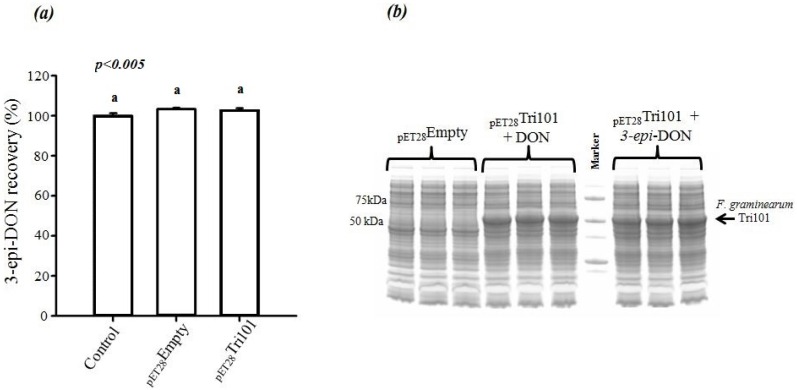
Changes in 3-*epi-*DON concentrations in *E. coli* BL21(DE3) cells overexpressing *Fusarium graminearum* Tri101. (**a**) No changes in 3-*epi-*DON recoveries were noticed for the construct that encoded the Tri101 enzyme in comparison to the control or empty vector. The above tendency was observed in two separate experiments, each with three replications, and the different letters signify significant differences between the means according to Fisher’s Least Significant Difference (LSD) test (*p <* 0.005); (**b**) To confirm that the above cells where all over-expressing His-FgTri101, bacterial pellets were collected, lysed, and ran on SDS-PAGE gels (4%–20%) before Coomassie Blue staining. The overexpression levels of His-FgTri101 were all evident in both DON and 3-*epi-*DON treatments.

On the other hand, incubation of 3-*epi-*DON with either *E. coli* BL21(DE3) cells expressing His-FgTri101 or BL21(DE3) cells with empty pET-28a(+) vector did not lead to any changes in 3-*epi-*DON recovery levels after methanol-extractions (Figure 4a) or to the detection of 3ADON presence in these reactions (data not shown) as a result of 3-*epi-*DON and FgTri101 interactions.

In order to assure that *E. coli* BL21(DE3) cells incubated with both DON and 3-*epi-*DON were all expressing FgTri101, a SDS-PAGE separation of total cell-lysates was conducted and the results are shown in Figure 4b. The three triplicates of DON/BL21(DE3)-FgTri101 cells expressed His-tagged FgTri101 in an equal fashion to 3-*epi-*DON/BL21(DE3)-FgTri101 cells offering the chance for both metabolites to interact with FgTri101 equally and forming 3ADON (if possible).

In an identical fashion, the in vitro reactions assembled as stated later within the Materials and Methods section yielded parallel observations (Table 1). No 3ADON was detected using LC-MS/MS analytical method when 3-*epi-*DON was incubated with purified his-tagged FgTri101 (tube #6) nor when either DON or 3-*epi-*DON were incubated with FgTri101 in the absence of acetyl-CoA (tubes #2 and #5). The 3ADON metabolite was only detectable in the presence of DON, FgTri101, and acetyl-CoA (tube #3).

### 2.4. 3-epi-DON Cross-Reactivity with DON-Monoclonal Antibodies Suggested Similar Structural Configurations/Side-Group Rearrangements of Both Compounds

A commercial monoclonal antibody that was generated against DON and is widely used to specifically capture this toxin from complex food matrixes/biological samples was tested for its ability to interact with 3-*epi-*DON. The assumption was that, if both compounds were captured and retained equally on the DONtest immunoaffinity column, then it is plausible to hypothesize that the two metabolites display similar epitopes and share close structural configurations needed for such monoclonal antibody recognition/interactions. In other words, despite the structural stress induced by the C3 carbon epimerization of DON (and the correlated electrons rearrangements to stabilize the resulting isoform), the two chemicals can be assumed to endure a similar epitope/3D chemical configuration in case they were both recognized/bound by the same monoclonal antibodies.

**Figure 5:**
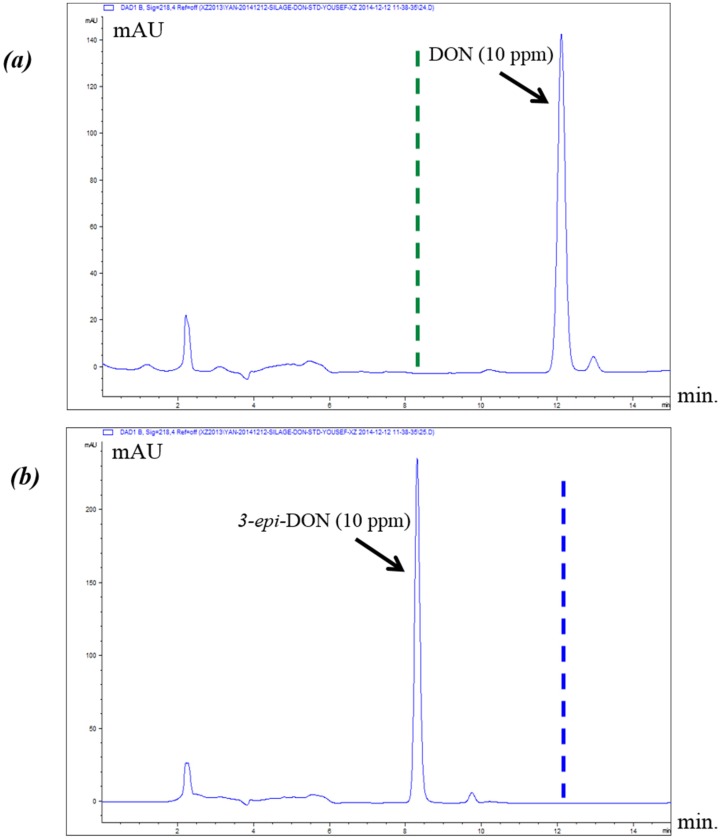
Both DON and 3-*epi-*DON interacted with the same conjugated commercial DON-monoclonal antibody in a similar fashion. The chromatographic separation of DON and 3-*epi-*DON after the affinity column purification step (DONtest) and injection on Jupiter 4µ Proteo 90A HPLC column is shown. The results indicated that DON (**a**) was captured in a similar fashion to 3-*epi-*DON (**b**).

Satisfyingly enough, the DONtest immunoaffinity column showed the ability to bind 3-*epi-*DON in a comparable fashion to DON. When the methanol eluted compounds were analyzed using HPLC methods described below, both metabolites were detectable as indicated in Figure 5.

The obtained results suggested that most of the 3D structural features of DON were still indeed conserved after carbon C3 epimerization. Such conserved features were responsible for the observed affinity between the tested DON-monoclonal antibody and 3-*epi-*DON in a similar fashion to the ones noticed earlier between DON with that exact monoclonal antibody.

### 2.5. Molecular Visualization of FgTri101/DON Interactions Highlighted the Role of Some Non-Polar Amino Acids

**Figure 6:**
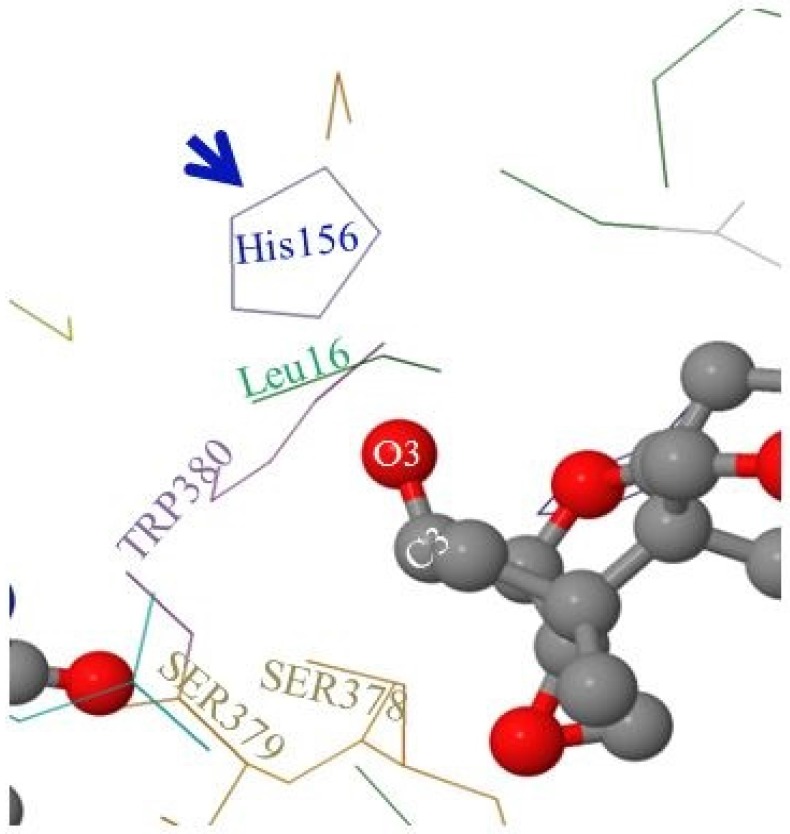
The molecular visualization of DON within *F. graminearum* Tri101 binding pocket. The C3 carbon (and attached -OH group) in DON (less-polar) is facing a non-polar amino acid (Leu16). In the case of 3-*epi-*DON (higher-polarity), the same group will be facing a matrix of non-polar (Leu16) and neutral (Trp380) amino acid residues.

As mentioned in the Materials and Methods section, the FgTri101 enzyme crystal structure was solved by Garvey et al. [28] showing the key amino acids that occupy the active pocket of the enzyme. Among these amino acids are: Leu16, TRP380, SER378, SER379, and the catalytic His156 (Figure 6).

When DON occupies the enzyme’s active pocket (and taking the steric hindrance in consideration), the C3-O3 group extends to establish the interactions with the nearby Leu16 and His156 and shows higher affinity toward the non-polar Leu16 residue (Figure 6). In contrast, when 3-*epi-*DON occupied the same space: (a) first, it elicited a reduce affinity toward Leu16 (an extension to the previously observed chromatographic behavior on the reverse phase column) and (b) second, the C3-O3 bond was slightly leaning away in the opposite direction. Altogether, this leads to decreased dwelling/interaction times between FgTri101 and 3-*epi-*DON in comparisons with the original interactions noticed for FgTri101 and DON. Such reduced interactions between 3-*epi-*DON and other molecular targets (including ribosomal binding sites) were reported to be the force that dictates 3-*epi-*DON reduced toxicity [12].

Furthermore, the molecular modeling and visualization of 3-*epi-*DON and FgTri101 interactions indicated while this metabolite do indeed fit within the Tri101 active site without any major conformational perturbations, the 3-OH group in 3-*epi-*DON is sufficiently disoriented to prevent the acetylation reaction from proceeding.

## 3. Discussion

Food and feed research is driven nowadays by a strong support from current consumers to adapt green biotechnological solutions [21,30]. The field is increasingly exploring new avenues within the bioremediation arena to mitigate the growing problems of mycotoxin contamination. DON presence in the food/feed chain is still considered as a challenging task to address as no effective biological means of control are optimized yet. Most recently, a number of research teams have reported the use of bacterial isolates to bio-transform DON to other metabolites with a significant reduction in the associated toxicity [2,18,31,32,33,34]. Among the reported bacterial metabolites within this regard were DOM-1, 16-hydroxy-deoxynivalenol, and 3-*epi-*DON. A clear mechanistic understanding of the phenomena behind the diminished toxicity of such metabolites is a must in order to avoid any unpredicted scenarios such as the case of previously reported masked mycotoxins [35].

To fully comprehend the reduction of DON toxicity upon C3 carbon epimerization [24,26,33], we tested the ability of 3-*epi-*DON to adapted/interact with the active pocket of a recombinant Tri101 enzyme cloned from *F. graminearum*. The Tri101 enzyme which represents a large group of acetyltransferases that naturally interact with DON, was expressed and purified in an exogenous host, namely *E. coli* BL21(DE3). The ability of the recombinant FgTri101 enzyme to transfer acetyl groups from acetyl-CoA to DON was used in this study as an indicative of such interactions while the absence of acetylated by-products, judged by LC-MS/MS analysis, hinted toward aberrant enzyme-substrate interactions. Other parameters including substrate polarity and 3D structural rearrangements were investigated too.

The obtained observations here collectively supported the highlighted role of C3 carbon in establishing the molecular interactions of DON with different cellular targets. This carbon plays a pivotal role in DON toxicity which emerges mainly from DON’s ability to bind to ribosomal RNA hence inhibiting protein bio-synthesis and suppressing host-defense mechanisms. A decent amount of scientific literature was dedicated for understanding the multifaceted relations between DON interactions with cellular targets including ribosomes, and overall bio-toxicity [2,9]. The conserved role of C3 carbon (modification and orientation) in DON bio-potency is not surprising. In fact, some of the mitigation approaches that were suggested earlier to reduce DON efficacy as plant immuno-inhibitor where to enzymatically modify this group through acetylation and/or glycosylation [27,36,37].

By far the most prominent interaction between DON and the PTC is mediated by the 3-OH group, which coordinates a structural Mg-Ion. Modeling 3-*epi-*DON/PTC shows that the epimerized 3-OH group is not in place to form reasonable interactions, and the strongest interaction (with the Mg-Ion) is broken. While the epoxide group is still in close contact with U2873 of the rRNA but due to the isomeric changes, 3-*epi-*DON cannot establish any bonds with U2869 [12].

In Tri101, 3-*epi-*DON could be modelled quite convincingly into the DON binding site, and there were no obvious clashes. The epimerized 3-OH group was out of reach for acetylation. This in addition to the increased polarity clearly shows why Tri101 cannot work with 3-*epi-*DON as a substrate.

Furthermore, the obtained data indicated that 3-*epi-*DON was still capable of interacting with a commercial DON-specific monoclonal antibody rolling out the assumption that a mere steric hindrance/global 3D structural rearrangements are the base for the observed reduced interactivity (and toxicity) associated with C3 epimerization. Collectively, the provided data suggested that the reduced toxicity of 3-*epi-*DON is due to a combination of reduced interactions and increased polarity index which both affect the observed interactions of 3-*epi-*DON at the molecular level as noted also by Pierron et al. [12].

The reported differences in polarity can be advantageous from the analytical point of view as they allow to validly distinguish between the two stereoisomers based on their separation patterns. The interactions of DON and 3-*epi-*DON with reverse phase HPLC columns packed with non-polar stationary phases clearly can be utilized to analyze both metabolites under isocratic conditions. The retention times of less-polar eluents (DON in this case) are substantially longer than the more-polar molecules (such as 3-*epi-*DON).

Since chemical modifications do in some cases affect the ability of certain chemicals of crossing cellular membranes as in the case of 15-acetyldeoxynivalenol (15ADON) which shows a higher permeability in human Caco-2 intestinal cells in comparison to DON or 3ADON [38] and in order to minimize the chances of aberrant interpretations, we used both in vitro and in vivo approaches to address the molecular intractability questions with Tri101. Both models yielded identical outcomes.

In conclusion, the presented work here provides the base for a better understanding of the correlations between DON, 3-*epi-*DON, role of C3 carbon epimerization, and host-toxicity at the molecular level. Further studies that securitize the effect of C3 carbon epimerization on the transport/absorbability of 3-*epi-*DON in polarized eukaryotic cells (such as Caco-2) can also provide great insights about the kinetics of this microbial metabolite in animal/human gastrointestinal tracts.

## 4. Materials and Methods 

### 4.1. Bacterial Strains, Plasmids, and Chemicals

LB broth (Cat. #244620) was obtained from BD (Franklin Lakes, NJ, USA). Kanamycin (Cat. #60615) and acetyl coenzyme A sodium salt (Cat. #A2056) were ordered from Sigma-Aldrich (Oakville, ON, Canada). Deoxynivalenol (Cat. #51481-10-8) was obtained from TripleBond (Guelph, ON, Canada) and stored at −20 °C freezer until usage. 3-*epi-*DON was prepared and purified as described earlier [39]. BL21(DE3) competent cells (Cat. #69450-3) and pET-28a(+) vector (Cat. #69864-3) were both purchased from Novagen-EMD Millipore (Etobicoke, ON, Canada). 

### 4.2. Recombinant F. graminearum Tri101 Acetyltransferase Cloning, Expression, and Purification

**Figure 7:**
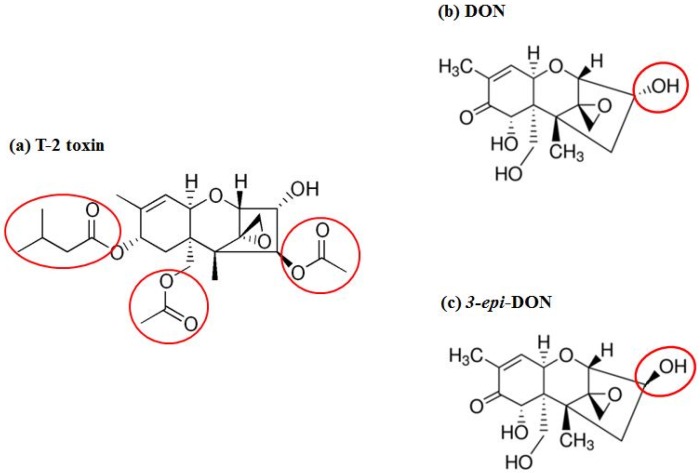
T-2 (**a**) and Deoxynivalenol (DON) (**b**) toxins were both reported to complex with FgTri101 despite the bulkiness of T-2 toxin in comparison to DON [28]. Due to the noted plasticity of FgTri101 active pocket, it is logical to assume that 3-*epi-*DON (**c**) can also bind within the enzyme’s catalytic cavity without any major structural restrain.

*Fusarium graminearum* Tri101 acetyltransferase (FgTri101) (GenBank # AB000874, 1356 bp) was selected to investigate DON and 3-*epi-*DON interactions for multiple reasons: (a) first, substrate-enzyme interactions can be measured indirectly through the determination of the acetylated-C3-DON in proportion to the un-modified original toxin; (b) second, the enzyme crystal structure was solved earlier by Garvey et al. [28] and clear insights about DON-binding site and the catalytic mechanism are already established; (c) third, the reported FgTri101 enzyme shows structural plasticity, particularly related to substrate-binding within the catalytic pocket. This plasticity is manifested by the ability of T-2 toxin to bind to the same site where DON was shown to bind [28]. Based on the bulkiness of T-2 structure (Figure 7a) (type A trichothecene) in comparison to DON or 3-*epi-*DON (Figure 7b,c), it can be logically assumed with confidence that steric hindrance that results from DON to 3-*epi-*DON epimerization should not be the sole factor that dictates 3-*epi-*DON interactions with FgTri101.

**F**gTri101 was cloned in pET-28a(+) vector after linearizing the vector with *NdeI* and *BamHI* restriction digests. gBlock fragments encoding FgTri101 sequence (optimized for *E. coli* expression) were ordered from IDT-DNA (Integrated DNA Technologies, Inc.; Coralville, IA, USA) and In-Fusion cloning reactions (In-Fusion HD Cloning Plus CE; Cat. #638916; TaKaRa-Clontech; Mountain View, CA, USA) were used to assemble the fragments according to the supplier’s protocol. His-tag was fused to the enzyme’s N-terminus. The resulting pET-28a(+)-His-FgTri101 kanamycin resistant vector was transformed into TOP10 competent cells (Life Technologies Inc., Burlington, ON, Canada) and selected on *Kan+* plates before sending for sequencing at the University of Guelph Laboratory Services.

The sequence-verified plasmid, pET-28a(+)-His-FgTri101, was transformed into *E. coli* BL21(DE3) competent cells which were grown at 37 °C for 5 h (OD_600_= 0.6–0.8) then transferred to 300 mL LB broth supplemented with kanamycin before inducing overnight with 0.5 mM IPTG (as final concentration). The induced culture was kept at 16 °C and 150 rpm until harvesting by spinning at 4500 rpm in swing-bucket type centrifuge. Collected pellets were subjected to a gentle protein extraction protocol using Qproteome bacterial protein preparation kit (Cat. #37900; Qiagen, Toronto, ON, Canada) according to the supplier’s recommendations. The His-tagged enzyme was purified by binding overnight to HisTALON Gravity columns (Cat. #ST0165; TaKaRa-Clontech), washing, and elution in 1 mL buffer increments. The purified enzyme was sub-aliquoted and stored at −20 °C until usage.

### 4.3. The in Vivo Acetyl-Transfer Assays

In the in vivo assays, *E. coli* BL21(DE3) cells either harboring empty pET-28a(+) or pET-28a(+)-His-FgTri101 vectors were incubated with either DON or 3-*epi-*DON separately. The resulting 3-acetyl-DON (3ADON) formation (if any) and reductions of DON/3-*epi-*DON concentrations were tracked using LC-MS/MS approach as described later. In short, tubes (in triplicates) containing LB broth supplemented with 50 ppm DON or 50 ppm 3-*epi-*DON were inoculated with 50 µL of overnight cultures of *E. coli* BL21(DE3) cells either harboring empty pET-28a(+) or pET-28a(+)-His-FgTri101. The cultures were kept growing on an orbital shaker (150 rpm) at 37 °C for 5 h (culture OD_600_ = 0.6–0.8) then were induced using IPTG (0.5 mM as final concentration) at 16 °C for another 16–20 h before spinning at 10,000 rpm and collecting the depleted broth and bacterial pellets for later analysis. Bacterial pellets were subject to SDS-PAGE analysis and Coomassie Brilliant Blue staining to confirm protein induction/expression while the depleted culture broth was extracted with 95% HPLC-grade methanol, filter-sterilized, and used as described later for DON, 3-*epi-*DON, and 3ADON analysis.

### 4.4. The in Vitro Acetyltransferase Enzymatic Assay

The purified His-FgTri101 enzyme prepared as described above was used as a model cellular target to investigate DON and 3-*epi-*DON in vitro interactions. In essence, working reaction mixes were prepared as suggested by Garvey et al. [28] through combining acetyl-CoA (0.62 mM as final concentration), 100 µg/mL DON or 3-*epi-*DON, 3.25 µg of His-FgTri101 enzyme in 50 mM Tris-HCl buffer (pH = 8). Mixes (*n* = 5) were kept at 28 °C overnight and reactions were terminated by adding absolute methanol in an equal volume. DON, 3-*epi-*DON, and 3ADON concentrations were measured using LC-MS/MS as described below. Both DON and 3-*epi-*DON recovery percentages were calculated in addition to 3ADON accumulation (if any).

### 4.5. Using Immunoaffinity Binding to Delineate DON and 3-epi-DON Structural Overlaps

Monoclonal antibodies can characteristically recognize a single, specific epitope that is displayed on a protein or a chemical. Such highly specific antibodies can be used generally speaking to track structural changes [40]. In the following approach we made use of this concept to tentatively test the ability of 3-*epi-*DON to bind to the same commercial monoclonal antibody generated against DON in order to gain insights about any structural changes associated with the epimerization of C3 carbon. An immunoaffinity column, DONtest HPLC obtained from VICAM (Cat. #G1005; MA, USA), packed with a DON-specific proprietary monoclonal antibody was tested for the ability to bind 3-*epi-*DON. In short, 1 mL of DON standard (1 μg/mL), 3-*epi-*DON (1 μg/mL), or combinations were loaded onto the pre-set columns and pressure was applied to achieve 1–2 drops/second flow rates. Columns were then washed with 5 mL of pure water and eluted with 1 mL of HPLC-grade methanol according to the supplier’s recommendations. The eluted DON and 3-*epi-*DON were dried either under nitrogen-streams or by using a SpeedVac concentrator (SPD2010, Thermo Scientific, Waltham, MA, USA) to be re-suspended later in 0.5 mL of 10% acetonitrile in water. Finally, samples were passed through 0.45 μm filters (Whatman, Florham Park, NJ, USA; Cat. #6765-1304) and analyzed using HPLC as described later.

### 4.6. HPLC Analysis

An Agilent HPLC system (1200 Series; Palo Alto, CA, USA) equipped with a quaternary pump, an inline degasser, an auto-sampler, and a diode array detector (DAD) was used in this study. A C12 reverse phase Phenomenex Jupiter 4µ Proteo 90A column (250 × 4.6 mm, Cat. #00G-4396-E0) coupled with a C18 guard column (Torrance, CA, USA) was used for the reported separation. A mobile phase consisting of two solvents (A: acetonitrile and B: H_2_O) mixed in 1:9 ratio by volume was used for elution. Isocratic conditions with flow rates controlled at 1 mL/min. were utilized with an injection volumes set to 50 μL. The total running time was 15 minutes while the detection wavelength was set at 218 nm.

### 4.7. LC-MS/MS Determination of DON and 3-epi-DON

Since mass spectrometry (MS) detectors have much better sensitivity levels, it was the preferred method to track DON, 3-*epi-*DON, and their acetylated products (if any). Liquid chromatography-tandem mass spectrometry (LC-MS/MS) was used to simultaneously determine DON and 3-*epi-*DON in methanol-extracted samples similar to what was described earlier by Young et al. [19]. In short, the system was composed of a Shimadzu HPLC system (Shimadzu, Kyoto, Japan) equipped with an UV/VIS photodiode array detector connected to a triple quadrupole IONICS 3Q Molecular Analyzer (IONICS, Bolton, ON, Canada). An Agilent (Agilent Technologies, Santa Clara, CA, USA) ZORBAX SB-C18 column (2.1 × 100 mm, 3.5 µm) was used for separation. Two mobile phases, solvent A (99.9% H_2_O + 0.1% formic acid) and solvent B (99.9% MeOH + 0.1% formic acid) were used. The chromatographic elution conditions were as the following: 0−1 min, isocratic 10% B; 1–10 min, gradient 10% to 80% B; 10–12 min, isocratic 80% B; 12–13 min gradient 80% to 10% B; 13–18 min, isocratic 10% B. The column temperature was set to 25 °C, the flow rate was controlled at 0.4 mL/min, and the injection volume was 10 µL, respectively. The ESI positive mode was used for data collection. Before carrying out the actual analyses, the system was optimized using standards at very low concentrations. Quantification was accomplished at multiple reaction monitoring (MRM) mode by monitoring the transition pairs of *m*/*z* 296.52 (molecular ion)/ 249.13 (fragment ion) for DON and 3-*epi-*DON and 338.83/231.13 for 3ADON. The fragment ion of each compound was selected based on the ion which has the highest sensitivity among the fragment ions of that compound. The dwelling time for MRM data collection was 200 ms. Peak areas obtained at the MRM mode were used to establish the calibration curves through least-squares regressions with R^2^ values ≥ 0.99.

### 4.8. Statistical Analysis and Molecular Visualization

All statistical analyses were conducted using SigmaPlot (version 12.5, Systat Software, Inc.; San Jose, CA, USA). One-way analysis of variance (ANOVA) was applied to the reported results followed by Fisher’s Least Significant Difference (LSD) test. Crystal structures of *F. graminearum* TRI101 complexed with Coenzyme A, Deoxynivalenol (#3B2S) and T-2 mycotoxin (#2RKV) were obtained from RCSB-Protein Data Bank. Both PyMOL (The PyMOL Molecular Graphics System, Version 1.7.4; Schrödinger LLC; Cambridge, MA, USA) and JSmol (https://sourceforge.net/projects/jsmol/) were used to visualize and manipulate the 3D structure of the reported complexes. 

## Figures and Tables

**Table 1:** The in vitro testing of FgTri101 ability to interact with DON and 3-*epi-*DON, respectively. Reaction mixes were assembled in triplicates as mentioned under the Materials and Methods section. Methanol-extracted aliquots were analyzed for 3ADON using the LC-MS/MS approach. Tri101 supported the acetylation of DON but not 3-*epi-*DON.

Component/Tube	(1)	(2)	(3)	(4)	(5)	(6)
DON	+	+	+	−	−	−
3-*epi-*DON	−	−	−	+	+	+
recombinant His-FgTri101	−	+	+	−	+	+
acetyl-CoA	−	−	+	−	−	+
3ADON formation/detection	No	No	Yes	No	No	No
